# The COVID-19 Pandemic and Hispanic/Latina/o Immigrant Mental Health: Why More Needs to Be Done

**DOI:** 10.1089/heq.2022.0041

**Published:** 2023-01-09

**Authors:** Cameron K. Ormiston, Jolyna Chiangong, Faustine Williams

**Affiliations:** Division of Intramural Research, National Institute on Minority Health and Health Disparities, Bethesda, Maryland, USA.

The United States (U.S.) Hispanic/Latina/o population comprises individuals of Cuban, Mexican, Puerto Rican, South American, Central American, and/or other Spanish culture or origin, regardless of race, and those of Latin American descent.^1,2^ Hispanic refers to individuals who are from or have ancestral origins from a Spanish-speaking country, whereas Latina/o is a pan-ethnic term that refers to individuals from >20 countries.^3^ Recently, Latinx has been used by popular culture and researchers to be more gender expansive, neutral, and inclusive, however, only 2–4% of Hispanics/Latinos report using the term.^4^

For the purposes of this editorial and based on recommendations from existing literature, we will use Hispanic/Latina/o and Latinx interchangeably according to what is used in the cited article and to be inclusive of all genders.^3,4^ As of 2021, 62.1 million Hispanics/Latinos reside in the U.S., and roughly a third of them were born in another country.^5^ People of Mexican origin make up a majority of the Hispanic/Latina/o population (59.5%), with Puerto Rican (9.3%), Salvadorean (4.0%), Cuban (3.8%), and Dominican (3.8%) being the next largest heritage groups.^5^

The median household income of Hispanics is $49,010, and an estimated 19% of Hispanics live in poverty, 59% have a high school degree or less, and 47% are homeowners. These numbers vary greatly by heritage group, underlining the heterogeneity of the U.S. Hispanic/Latina/o population.^3^ In addition, health disparities persist compared with non-Hispanic whites with Hispanics having higher rates of poverty, liver disease mortality, and uninsured individuals as well as being disproportionately affected by obesity and diabetes.^6^

The COVID-19 pandemic has especially laid bare the health inequities affecting Hispanic/Latina/o immigrants, with recent reports indicating Hispanics have the highest age-adjusted infection rates than all other racial/ethnic groups, and COVID-19 cases are higher in areas with a larger proportion of Hispanics, undocumented individuals, and immigrants.^7,8^ Furthermore, Hispanics are at the highest risk for SARS-CoV-2 infection, hospitalization, and mortality.^7^ Hispanic/Latina/o immigrants are also more likely to have low-paying jobs and live in lower income neighborhoods and overcrowded housing,^7,9^ which increase the risk for COVID-19 transmission.

Hispanic immigrants—particularly noncitizens—face numerous structural and health inequities that can increase COVID-19 vulnerability, such as diminished health care access due to citizenship status, fear of deportation, stigma, and exclusion from social program eligibility.^7,10,11^ These structural barriers coupled with heightened risk for COVID-19 exposure may increase risk of mental health symptoms and adverse mental health outcomes for Hispanic/Latina/o immigrants.^7,12,13^ More evidence, however, is needed on Hispanic/Latina/o immigrant mental health in the U.S. in relation to the effects of the COVID-19 pandemic.^12,14^

The pandemic's negative effects on mental health among the general U.S. population have been well documented, with psychological distress prevalence being significantly higher than prepandemic levels.^15,16^ Moreover, immigrant populations experience socioeconomic and structural vulnerabilities that can negatively impact mental health.^17^ Indeed, Hispanics were disproportionally affected by the pandemic-induced recession in the spring of 2020, in part reflecting their over-representation in some of the hardest hit sectors of the economy.^18^

According to the U.S. Bureau of Labor Statistics, in 2020, the unemployment rate for Hispanics was 10.4%, as compared with 7.5% for non-Hispanic whites.^19^ In addition, among full-time year-round workers in 2020, the average Hispanic/Latina/o median household income was $55,321 in comparison with $74,912 for non-Hispanic white households, which showcase an alarming socioeconomic disparity.^19^ Therefore, understanding that Hispanic immigrant populations face unique vulnerabilities, there is potential that they may experience a high burden of COVID-19-related adverse mental health outcomes.^12^

The Social Determinants of Health (SDH) provide a framework that can be utilized to categorize and explain the disparities in mental health outcomes experienced in the Hispanic/Latina/o community during the COVID-19 pandemic. Indeed, the link between mental health outcomes and SDH is well documented among Hispanic/Latina/o immigrants.^20–22^ The U.S. Department of Health and Human Services defines SDH as the conditions in the environments where people are born, live, learn, work, play, worship, and age that affect a wide range of health, functioning, and quality-of-life outcomes and risks.^23^

These determinants can be further grouped into five domains: economic stability, education access and quality, health care access and quality, neighborhood and built environment, and social and community context.^23^ For this editorial, we have chosen to focus on the domains of economic stability, neighborhood and built environment, and health care access and quality as we conceptualize and delve into the adverse mental health experiences of Hispanic/Latina/o immigrants during the COVID-19 pandemic.

## Economic Stability

Economic instability, financial stress, and worry are associated with psychological distress.^24–26^ Notably, Hispanic immigrants are more likely to be frontline, low-wage, and uninsured workers,^7,10,11^ which provides additional financial mental health stressors during the COVID-19 pandemic. Furthermore, there is evidence of a pandemic-related economic downturn disproportionately affecting Hispanic immigrants, especially noncitizen immigrants.^10,12^ For example, Hispanic immigrant families with at least one noncitizen have experienced higher rates of job loss, difficulties in paying bills, and food insecurity due to the pandemic.^10^

Social insecurity and economic and employment difficulties have been previously identified as risk factors for psychological distress among immigrant communities.^12^ The inequitable impact of these economic fallouts likely places Hispanic immigrants at risk for negative mental health consequences. For instance, Serafini et al^12^ reported a significant association between days being unable to work and psychological distress among undocumented Hispanic immigrants during the pandemic (March 2020).

As previously mentioned, Hispanic immigrants are more likely to be essential workers and are less likely to have occupations that allow for virtual at-home employment, increasing the risk of infection and subsequently disease anxiety.^7,10,11^ For example, Hispanic immigrants disproportionately make up the agricultural, meatpacking, and service-based industries, where numerous COVID-19 outbreaks have been reported and in-person contact risk is higher.^7,10,11^

A study of Mexican immigrants living in Los Angeles and New York City found participants working as essential workers felt compelled to continue working out of fear of losing their job, even if a family member at home was COVID-19 positive.^7^ These situations may contribute to inequitable burden of adverse mental health among Hispanics/Latinos. In July 2020, the rate of U.S. adults seriously considering suicide was highest among Latinx and essential workers.^13^

## Neighborhood and Built Environment

The physical environment is widely acknowledged to be a determinant of health, and factors such as crowded living and poor housing quality have been noted to negatively impact mental health.^27^ Hispanic immigrants are more likely to live in overcrowded and/or intergenerational households compared with other racial/ethnic groups.^7^ This makes quarantining, social distancing, or self isolating from other members difficult and may increase COVID-19 transmission risk, especially if there is an essential worker or positive case in the household.^7^ These living situations may in turn increase disease anxiety.^7,9^

In addition, COVID-19 containment measures, although important, may be detrimental to Hispanic/Latina/o immigrant mental health. For instance, stay-at-home orders and travel bans may have isolated immigrants from their support systems in their local community and host country, which could exacerbate mental health issues.

## Health Care Access and Quality

According to Serafini et al's^12^ March 2020 study of Hispanic immigrant outpatients in New York City, 50% reported worsened depression/anxiety symptoms during the pandemic compared with prepandemic symptoms. Furthermore, they found 60% of patients had serious mental illness.^12^ A national sample of U.S. adults also found Hispanic participants were significantly more likely to report not only fears and worries about COVID-19 than non-Hispanics, but also anxiety and depressive symptoms than whites.^15^

Unfortunately, initial reports from August 2020 to February 2021 indicated Hispanics/Latinos have a significantly higher unmet need for mental health services during the pandemic than other racial/ethnic groups.^14^ They also face an inadequate number of mental health providers and services, especially with heightened demands during the pandemic, transition to telehealth, and barriers to care for individuals with limited or no internet access.^7,9,14^ These issues are not new, however, as insufficient and inaccessible mental health services for immigrants existed before the pandemic due to poor infrastructure for affordable and linguistically accessible and culturally competent services.^7,9,14,28^

Moreover, care access issues during the pandemic may be further compounded by the already low mental health utilization by Hispanic/Latina/o immigrants before the pandemic due to inadequate awareness of services and how to access them, sociocultural factors, stigma, financial constraints (i.e., lack of insurance), immigration status, discrimination, and language barriers.^7,9^

In addition, both undocumented and documented Hispanic immigrants have reported fears of immigration authorities that is heightened by threat of surveillance, policing, or deportation.^7,29^ They have also reported fear of acquiring a public charge through the Public Charge Rule (e.g., rule that imposes sanctions: revocation or denial of visas, precluding the right to sponsor a family member for immigration, or even deportation, for lawful permanent residents who are eligible for or have utilized social programs in the past).^7,9,11,13,30^

Although the Public Charge Rule was overturned in March 2021, many immigrants are either unaware or residual fear still remains.^9^ Concerns about law enforcement and public charge have increased distress, mistrust in health systems, and reluctance toward accessing health services both before and during the pandemic.^7,9,11,13,30^ Owing to this mistrust and legal fears, Hispanic/Latina/o immigrants may not feel safe to reach out to mental health care providers, making them less likely to seek care and more likely to delay care, which in turn may worsen their mental health.^9^

## Solutions

A solution with roots in public health and medicine is warranted to formulate practitioner, research, and policy recommendations.

### Practitioner recommendations

Practitioners should continue to provide both telehealth and, when possible, in-person mental health services or a combination of the two services for patients. A study on undocumented Hispanic immigrants in New York City found >90% of participants agreed that the availability of remote psychiatry sessions helped manage their mental health.^12^ In addition, language services should always be available as the lack of language-concordant providers has been cited as a barrier to mental health care access.^9^ Although telehealth has shown potential in ameliorating barriers to mental health care utilization,^12^ practitioners should be wary of the digital divide and adapt to the needs of each patient.

For instance, Hispanic immigrants may be vulnerable to resource scarcity and may lack access and/or feel comfortable or safe utilizing virtual care services given concerns of confidentiality, cultural competency, language concordance, internet access, and lack of interpersonal connection with providers.^7,12^ Given many of these issues can be resolved through providing in-person care, it is suggested that telehealth should be provided on a case-by-case basis.^12^

In addition, practitioners must be educated on culturally humble, evidence-informed methods of care that recognize the unique heterogeneous experiences of Hispanic/Latina/o immigrants. In fact, culturally humble care may attenuate the adverse effects of negative provider encounters and can improve trust and patient satisfaction among Latinos.^31,32^ Moreover, Garcini et al's^13^ community-based study of community health workers (CHWs) found emphasizing family, religious, and social support through peer groups, as well as culturally relevant cognitive approaches (i.e., collectivist thinking) were effective ways to limit distress during the pandemic among Latino immigrants along the U.S.–Mexico border.

Also, consideration and integration of Latino cultural values such as *respeto* (mutual and reciprocal respect), *controlarse* (self-control of negative affect), *aguantarse* (ability to survive stressful situations during hard times), *familismo* (importance of family), *personalismo* (personal relationships), *confianza* (relationship trust), *fatalismo* (fatalism), *dichos* (popular sayings), and *sobreponerse* (self-suppression) into care plans and therapeutic alliances can also assist in creating more culturally informed mental health care plans.^33,34^ Importantly, clinicians must be educated in immigrant-specific processes, such as acculturation.

Having CHWs and *Promotor/as* (Latino community members trained to provide health education and resources to the local community) be actively involved in public health interventions may also be effective.^13^ For instance, the relatability and comfortability of community members within health care settings can assist in wearing down barriers between Hispanic/Latina/o patients and health care providers by providing culturally adapted and relevant education and strengthening trust.^35^ Previous research has highlighted the positive impacts CHWs can have on both the physical and mental health of Hispanics/Latinos given their unique position in their respective communities.^35,36^ Bolstering community care centers with trained medical translators and interpreters is also essential.^9,12,13^

### Research recommendations

Our understanding of health inequities and disparities is often based on how data are interpreted and disseminated. Research and data collection, therefore, must be improved to better capture the diverse situations and needs of Hispanic/Latina/o immigrants. For example, the Hispanic/Latina/o immigrant population is often analyzed as an aggregated group,^9^ which obscures several differences across heritage groups, region of residence, intersectional identity, as well as immigration and generational status. Through disaggregated data collection, interventions can be more culturally specific and tailored to the specific needs of the local immigrant community.

Moreover, national data sets and cross-sectional surveys are often not culturally adapted to diverse communities, particularly with the health assessments and questionnaires used in national surveys in addition to conceptualizations and interpretations of findings. Data collected among these communities subsequently may not reflect the actual lived mental health experiences.^37^ Thus, qualitative and longitudinal studies that utilize primary data are warranted. There is also a need for ongoing research to address the long-term effects of the pandemic on Hispanic/Latina/o mental health. Ultimately, research and data should be used to directly benefit the study population—Hispanic/Latina/o immigrants—by informing policy and public health interventions to mitigate the structural and systemic barriers to mental well-being.

### Policy recommendations

The COVID-19 pandemic has particularly exacerbated the unmet needs and social insecurity of undocumented Hispanics, with >50% reporting difficulty paying for their rent, food, and utilities.^12^ Alarmingly, these numbers may continue to increase given the long-term effect of the pandemic on health and well-being, and most especially since some immigrants were not eligible for stimulus packages despite being essential contributors to the U.S. economy.^9,12,38^ Living in mixed-status households (households with individuals of variable immigration status) may also cause delays in accessing or/forgoing mental health services due to fear of bringing attention to undocumented members of the household.^9^

Breaking down structural barriers by (1) including undocumented individuals in government stimulus packages, (2) providing greater free and/or affordable mental health resources to immigrant populations, and (3) creating an environment where immigrants have equitable access to health care without fear of immigration authorities or repercussions is paramount.

Wide-scale mental health screening should be initiated especially within Hispanic/Latina/o immigrant communities with limited access to services and/or resources. However, these initiatives require public and private funding for the necessary workforce and support of social workers, psychiatrists, and other trained mental health care providers. Furthermore, investment should also be put into CHWs to help educate Hispanic/Latina/o immigrants on current mental health resources, how to access them, and engage in dialogue to dispel fears regarding the use of mental health resources, seeing that this has served as a barrier.

## Conclusion

In summary, researchers, policymakers, and practitioners should directly engage with stakeholders and members of all Hispanic/Latina/o immigrant heritage groups, generational status, and intersectional identity.^9^ By doing so, we can work toward preventing the harmful long-lasting impacts of the COVID-19 pandemic on the mental health and well-being of the fastest growing immigrant subpopulations in the United States.

## Abbreviations Used

CHWs: community health workers
SDH: Social Determinants of Health
